# Large Isoform of Mammalian Relative of DnaJ is a Major Determinant of Human Susceptibility to HIV-1 Infection

**DOI:** 10.1016/j.ebiom.2014.10.002

**Published:** 2014-10-07

**Authors:** Yu-Ping Chiang, Wang-Huei Sheng, Pei-Lan Shao, Ya-Hui Chi, Yi-Ming Arthur Chen, Szu-Wei Huang, Hsiu-Ming Shih, Luan-Yin Chang, Chun-Yi Lu, Shan-Chwen Chang, Chien-Ching Hung, Li-Min Huang

**Affiliations:** a Department of Pediatrics, National Taiwan University Hospital and National Taiwan University College of Medicine, Taipei 100, Taiwan; b Department of Internal Medicine, National Taiwan University Hospital and National Taiwan University College of Medicine, Taipei 100, Taiwan; c Institute of Biotechnology and Pharmaceutical Research, National Health Research Institutes, Zhunan 350, Taiwan; d Center for Infectious Diseases and Cancer Research (CICAR), Kaohsiung Medical University, Kaohsiung, Taiwan; e Institute of Biomedical Sciences, Academia Sinica, Taipei 115, Taiwan

**Keywords:** Human immunodeficiency virus type 1 (HIV-1), Macrophages, Mammalian relative of DnaJ (MRJ), Susceptibility to infection

## Abstract

Individual differences in susceptibility to human immunodeficiency virus type 1 (HIV-1) infection have been of interest for decades. We aimed to determine the contribution of large isoform of Mammalian DnaJ (MRJ-L), a HIV-1 Vpr-interacting cellular protein, to this natural variation. Expression of MRJ-L in monocyte-derived macrophages was significantly higher in HIV-infected individuals (n = 31) than their uninfected counterparts (n = 27) (p = 0.009). Fifty male homosexual subjects (20 of them are HIV-1 positive) were further recruited to examine the association between MRJ-L levels and occurrence of HIV infection. Bayesian multiple logistic regression revealed that playing a receptive role and increased levels of MRJ-L in macrophages were two risk factors for HIV-1 infection. A 1% rise in MRJ-L expression was associated with a 1.13 fold (95% CrI 1.06–1.29) increase in odds of contracting HIV-1 infection. Ex vivo experiments revealed that MRJ-L facilitated Vpr-dependent nuclear localization of virus. Infection of macrophage-tropic strain is a critical step in HIV-1 transmission. MRJ-L is a critical factor in this process; hence, subjects with higher macrophage MRJ-L levels are more vulnerable to HIV-1 infection.

## Highlights

• Our results suggest that individuals with high levels of MRJ-L may be more susceptible to HIV-1 infection than individuals with low levels of MRJ-L.
• We demonstrated that MRJ-L interacts with HIV-1 Vpr and assists with HIV-1 replication. A rise in MRJ-L levels effectively increases the replication of HIV-1 and a reduction in MRJ-L expression significantly decreases HIV-1 production.
• Strategies to lower MRJ-L levels in macrophages may be beneficial in controlling HIV-1 infection.

## 1 Introduction

More than 30 million individuals worldwide are currently infected with human immunodeficiency virus type 1 (HIV-1), the cause of acquired immunodeficiency syndrome (AIDS), with 1.7 million deaths occurring from AIDS in 2011 (World Health Organization, 2011). Currently, combination antiretroviral therapy is successful in controlling the disease progression of HIV infection. However, antiretroviral therapy is known to be associated with deleterious complications such as metabolic derangement (Paula et al., 2013). Cessation of antiretroviral therapy is not feasible as it may lead to viral rebound and disease progression (Costiniuk et al., 2013). Moreover, lifelong antiretroviral treatment poses a great economic burden to society as well as the family.

Alternative strategies to control HIV-1 infection are highly desirable. These may include manipulation of the immune response and other host factors. Prophylactic HIV-1 vaccines do not appear to show immediate promise, following the failure of a recent clinical trial (Cohen, 2007). A therapeutic HIV-1 vaccine has produced some positive results but still requires further development before it can be brought to the clinic (Pollard et al., 2014). Manipulation of host proteins is currently being evaluated, as demonstrated by a study to modify the CCR5 receptor (Didigu et al., 2014). This is a promising way to complement the current antiretroviral therapy. Nevertheless, its success relies on our understanding of the interaction between HIV-1 and cellular proteins.

It has long been of interest in the field of infectious diseases that different individuals show varying degrees of susceptibility to infection. This variation may be due to differences in host protein expression. For example, one of the most well-known susceptibility-determining factors for HIV infection is the CCR5 co-receptor. A deletion of 32 bp in the CCR5 receptor confers a much reduced susceptibility to HIV-1 infection for the host (Liu et al., 1996, Dean et al., 1996, Samson et al., 1996). A number of other enhancing and restrictive factors on HIV-1 replication have been proposed, including TRIM5α, APOBEC3 family, BST-2/tetherin, HLA-G, and microRNAs (Lever and Lever, 2011, da Silva et al., 2014, Napuri et al., 2013). However, whether they affect individual susceptibility to HIV-1 infection remains elusive (Imahashi et al., 2014).

Viral infection begins with the binding of a cellular receptor, followed by viral entry and replication. Many cellular factors interact with viral genes for the completion of the replication cycle and production of mature virions. We reasoned that cellular and viral protein interactions may contribute to varying degrees of susceptibility to viral infection. Hence, we examined Vpr-associated cellular proteins and studied their roles in human HIV-1 infection. In a previous study, by yeast-two-hybrid assay we identified several cellular proteins that interact with HIV-1 Vpr (Yedavalli et al., 2005). Of them, DNAJB6 is a homolog of heat shock protein 40 (HSP40), and has two splice isoforms: the large form MRJ-L and the small form MRJ-S (Hanai and Mashima, 2003). Prompted by the notion that DNAJ is a heat shock protein which may contribute to HIV-1 replication, we investigated the expression of DNAJB6 in lymphocytes and monocyte-derived macrophages (MDMs) which are considered the major targets of early HIV-1 infection.

## 2 Materials and Methods

### 2.1 Participant Recruitment and Serologic Screening for HIV Infection

Self-completed questionnaires and blood samples were collected from anonymous participants at commercial male homosexual venues of Taipei and New Taipei City on weekends. The questionnaires included measures of demographics, HIV testing history and sexual risk behavior (Supplementary Table 1). A total of 1200 study participants received pre-test counseling, were informed about the purpose of this study and gave written informed consent. HIV diagnosis was made using a recombinant HIV enzyme immunoassay (Murex Diagnostics Limited, Dartford, UK). Positive test results were confirmed by HIV western blot 2.2 (Genelab Technologies, Inc., Singapore). In addition, positive blood samples were tested for HIV-1 subtype. Based on the regular blood sampling every 3 months, 89 participants were infected with HIV-1. Of the subjects who were identified to be seroconverted within prior 6 months, 20 of them were recruited to join this MRJ-L evaluation study. Another 30 uninfected subjects were randomly recruited as controls. This study was conducted with the approval of the institutional review board of the Mackay Memorial Hospital, Taipei, Taiwan.

### 2.2 Cell Lines and Cell Preparations

After obtaining informed consent, CD4 + T lymphocytes and CD14 + monocytes were isolated from PBMCs from healthy donors and HIV-infected patients by Ficoll-Paque gradient (GE Healthcare Life Sciences, Pittsburgh, PA, USA) using CD4 +/CD14 + magnetic microbeads (Miltenyi Biotec, Bergisch Gladbach, Germany). Laboratory personnel were blind to the identity and HIV-1 infection status of blood donors. To induce macrophage differentiation, monocytes isolated from donors were maintained in RPMI1640 supplemented with 10% human AB serum, 5% fetal bovine serum (FBS), and 50 ng/mL M-CSF (PeproTech, Rocky Hill, NJ, USA) for seven days. Jurkat cells were cultured in RPMI1640 supplemented with 10% FBS. In addition, phytohaemagglutinin (PHA) was added to stimulate Jurkat cells. THP-1 (a monocyte cell line) and U937 cells were cultured in RPMI1640 (Life Technologies, Grand Island, NY, USA) supplemented with 10% FBS and 2 mM DMSO. 293 T cells were cultured in DMEM (Life Technologies) supplemented with 10% FBS. To induce differentiation of U937 cells into macrophage-like cells, 200 μM phorbol myristate acetate (PMA) mitogen was added to the culture medium, and the cells were harvested after 48 h.

### 2.3 Plasmids

The plasmid pEGFP–Vpr was constructed by inserting the Vpr gene amplified from the HIV-1 pNL4-3 clone into the appropriate vectors. The HA-MRJ-L and HA-MRJ-S plasmids were constructed in the pcDNA vector. The lentiviral expression vector of MRJ-L was constructed in the pLKO_AS3w.puro vector. The MRJ-L 3′UTR shRNA (shJ8_5′-GCGCAGATGGCTAACTGAGTA) was designed using InvivoGen siRNA Wizard v3.1 software, and was constructed in the pLKO.1-puro vector (obtained from the National RNAi Core Facility, Academia Sinica, Taiwan). All construct identities were confirmed by sequencing the entire insert.

### 2.4 HIV-1 Virus Production and Viral Infection

293 T cells (2 × 10^6^) were transfected with 2 μg of p125 (M-tropic, ADA strain) plasmids using Lipofectamine 2000, and the supernatants were harvested at 48 h post-transfection. Viruses were pretreated with 2 U/mL DNase I (Life Technologies) at 37 °C for 30 min before infection, and the viral titer was quantified using the p24 ELISA assay (PerkinElmer, Waltham, MA, USA). Cells with over-expressed or reduced levels of MRJ-L were titrated to adjust for equal cell numbers, and 1 × 10^6^ cells were infected with M-tropic strain of HIV-1 (equivalent to 25 ng of p24 antigen) at 37 °C for 2 h. After washing three times with PBS, cells were maintained in RPMI1640/2% FBS and half of the medium was replaced every three days. Culture supernatants were collected every three days for quantification of p24 antigen by HIV-1 p24 ELISA (PerkinElmer).

### 2.5 Statistical Analysis

The significance level was set at 0.05, and all p values were two-tailed. Risk factors for HIV-1 infection were analyzed using simple logistic regression, followed by Bayesian multiple logistic regression because of sparse data. The simple logistic regression was performed using the software Stata (version 13, StataCorp, College Station, Texas) or WinBUGS (version 1.4.3; MRC Biostatistics Unit, Cambridge, England) when there were sparse data. Bayesian multiple regression analysis was undertaken using the software package WinBUGS.

### 2.6 Ethical Research Conduct

This study conformed to the provisions of the 1975 Helsinki Declaration and was approved by the institutional review board of the National Taiwan University Hospital (NTUH-201102003RC) and Mackay Memorial Hospital (13MMHIS039). All patients gave written consents before they participated in this study.

## 3 Results

### 3.1 MRJ-L Expression in Macrophages Correlates With HIV-1 Susceptibility in Primary Human Cells

**Fig. 1:**
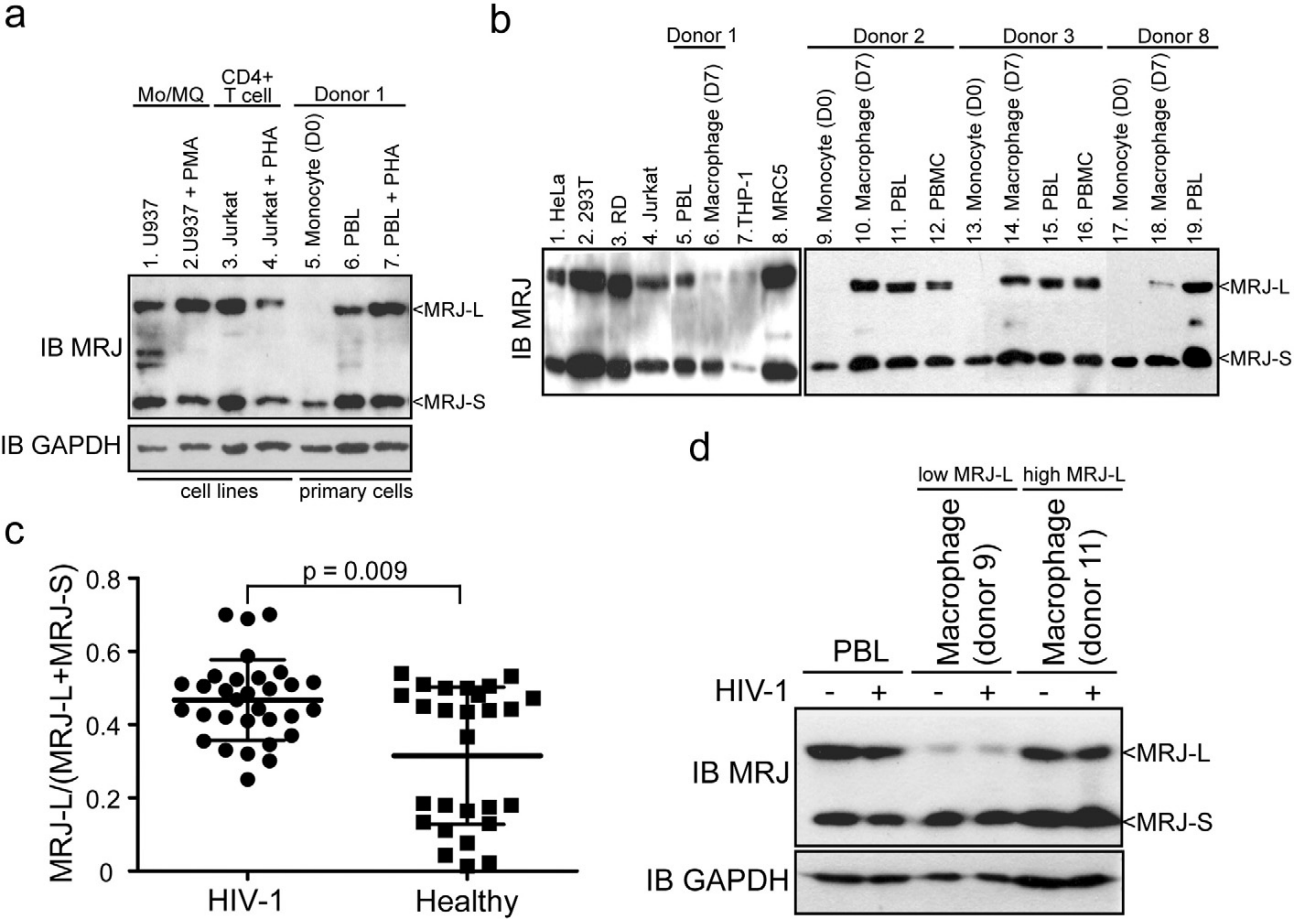
Expression of MRJ-L and MRJ-S in cell lines and donor PBMCs. (a) Western blot analysis of endogenous MRJ-L and MRJ-S in various cells. Significant amounts of MRJ-L and MRJ-S are seen in U937, PMA-stimulated U937, Jurkat and PHA-stimulated Jurkat cells (lanes 1–4). PBL (peripheral blood lymphocytes) and PHA-stimulated PBLs from donor 1 express similar amounts of MRJ-L and MRJ-S (lanes 6–7). Note, however, that monocytes (lane 5) express only MRJ-S, but no MRJ-L. GAPDH was used as a loading control. (b) Western blotting of MRJ-L and MRJ-S in cells from healthy donors and cell lines. (c) MRJ-L expression in macrophages from 31 HIV-1-infected patients and 27 healthy donors. Significantly higher levels of MRJ-L are expressed in macrophages from HIV-1-infected individuals than uninfected controls. Average ± SD is shown. p = 0.009 by the Mann–Whitney Rank Sum test. (d) HIV-1 infection does not alter the expression level of MRJ-L in PBL and macrophages from donor 9 (low MRJ-L) or donor 11 (high MRJ-L). Cells were harvested at day 8 post-HIV-1 infection. Expression of GAPDH was compared for normalization.

Based on data from cells of 27 uninfected and 31 HIV-1-infected donors [median age was 25 years (range, 19–30 years), median CD4 count was 371/μL (range, 276–656/μL) and median plasma HIV RNA load was 18,400 copies/mL (range, 904–242,000 copies/mL)] as well as from cultured T-cell lines, we noted that MRJ-S was found in all monocytes, macrophages, and cultured cell lines, while MRJ-L was uniformly absent from monocytes (Fig. 1a, b). Indeed, MRJ-L protein was absent in monocytes from all 27 uninfected donors, whereas macrophages derived therefrom had varying amounts of MRJ-L (see illustrative results from donors 1, 2, 3, and 8; and cultured cell lines in Fig. 1b). The MRJ-L expression level was defined as the ratio of MRJ-L divided by total MRJ (MRJ-L plus MRJ-S). Intriguingly, HIV-1-infected individuals, compared with uninfected controls, had statistically higher MRJ-L levels (p = 0.009 by the Mann–Whitney rank sum test; Fig. 1c).

There are two ways to interpret the finding that macrophages in HIV-1-infected individuals have higher MRJ-L levels than macrophages from uninfected individuals. One interpretation is that MRJ-L expression is stochastically variable among individuals and that those with higher MRJ-L levels are more susceptible to HIV-1 infection. This would explain the prevalence of high MRJ-L expressors among HIV-1-infected individuals. The second interpretation is that MRJ-L levels are constitutively low and that it is in fact infection by HIV-1 per se that increases MRJ-L expression. To distinguish between these two possibilities, we infected macrophages with HIV-1 and determined MRJ-L levels immediately after infection as well as eight days later (Fig. 1d). No significant changes in MRJ-L expression levels were seen in cells infected with HIV-1. This finding supports the interpretation that individuals with stochastically high MRJ-L expression are more susceptible to HIV-1 infection.

### 3.2 Increased Macrophage MRJ-L Expression is a Significant Risk Factor for HIV-1 Infection Among Men Who Have Sex With Men

To confirm our findings, we recruited a group of male cohort comprised primarily of men who have sex with men (MSM) (96%) with a mean age of 29 years (range 19–45 years). These study participants were negative for HIV antibody at baseline and underwent follow-up HIV testing every three months. Among the 1200 subjects being followed, 20 subjects who were identified to be seroconverted within prior 6 months were recruited to join this MRJ-L evaluation study. Another 30 uninfected participants were also recruited as controls. There were no significant differences between infected and uninfected individuals in regard to age, sexual orientation, marital status, occupation, education, number of sexual partners, frequency of sexual contact, recreational drug use, and frequency of condom use (Supplementary Table 1).

**Fig. 2:**
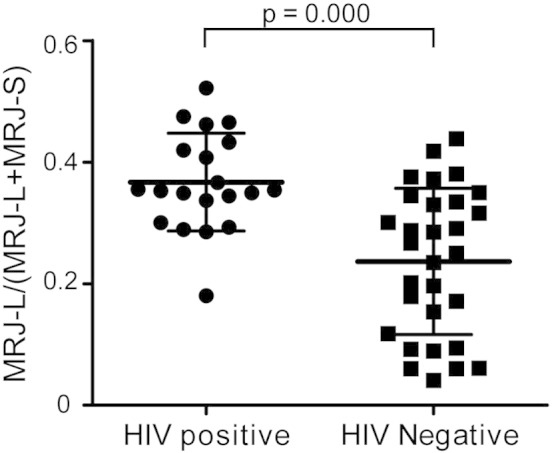
Expression of MRJ-L and MRJ-S in 50 MSM participants. Schematic presentation of MRJ-L expression in macrophages from 20 HIV-1 recent infected patients and 30 negative controls. Significantly higher levels of MRJ-L are expressed in macrophages from HIV-1 infected individuals (36.71 ± 8.05%) than uninfected controls (23.65 ± 12.04%). Average ± SD is shown. p = 0.000 by Mann–Whitney Rank Sum test.

Bayesian multiple logistic regression revealed that playing the receptive role in unprotected anal intercourse (RUAI) and MRJ-L levels (Fig. 2) were two independent significant risks for HIV-1 infection. After controlling for other factors, we determined that a 1% rise in MRJ-L expression was associated with a 1.13 fold (95% CrI 1.06–1.29) increase in odds of contracting HIV-1 infection. RUAI was linked to a 43.9 fold (95% CrI 2.7–2957) increased odds of HIV-1 infection compared with insertive unprotected anal intercourse (IUAI) (Table 1 and Supplementary Table 2). Because our data contain several zero cells (i.e. no subjects in those categories), this sparseness of data yielded large credible intervals in some analyses.

The distribution of MRJ-L levels in Figs. 1c and 2 showed a bimodal distribution in the HIV-1 naïve population. To access risks of the general population for HIV-1 infection, we defined the MRJ-L level as high if the ratio of MRJ-L divided by total MRJ was > 0.4 (one standard deviation above the average of HIV-1 infected and HIV-1 uninfected individuals, calculated based on the values in Fig. 2), as low if the ratio was < 0.25 (average of HIV-1 uninfected individuals), and as medium if the ratio was between 0.25 and 0.4. By these measures, of the 50 MSM participants, 35% of HIV-1 seropositive individuals showed high levels of MRJ-L expression in contrast to 7% of HIV-1 negative cohorts (Table 1 and Fig. 2). Using Bayesian logistic regression analysis, we found that subjects with medium levels of MRJ-L had a 13.8 fold (95% credible interval [CrI] 1.6–121.4) higher odds of contracting HIV-1 infection compared to those with low levels of MRJ-L. The odds increased to 52.5 fold (95% CrI 4–680.9) for subjects with high levels of MRJ-L. Bayesian multiple logistic regression (Supplementary Table 2) revealed subjects with medium levels of MRJ-L had a 7.6 fold (95% CI 1.4–69.2) higher odds of contracting HIV-1 compared with those with low levels of MRJ-L. The odds increased to 41.5 fold (95% CrI 4.3–705.7) for subjects with high levels of MRJ-L.

### 3.3 Macrophages With Low Levels of MRJ-L are Resistant to Infection With Low HIV-1 Inoculum

**Fig. 3:**
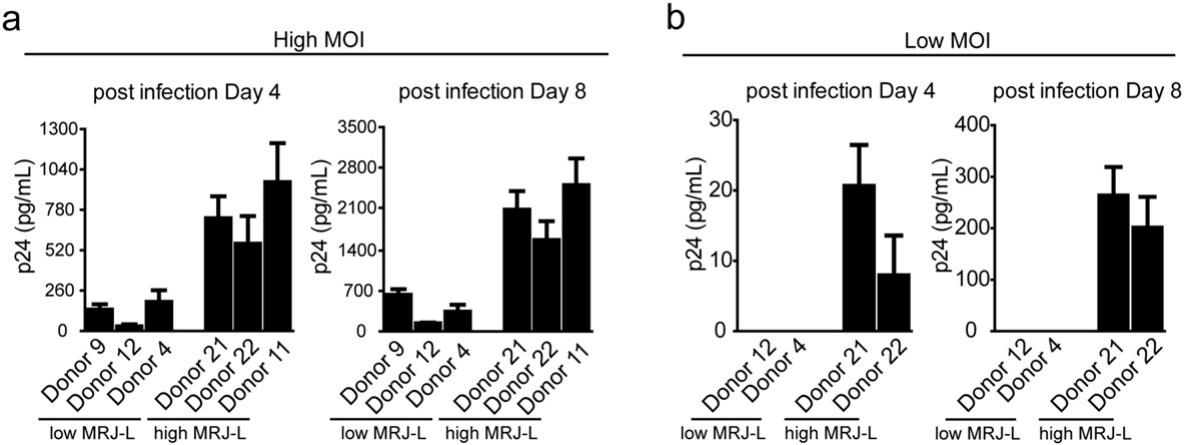
Expression levels of MRJ-L correlate with HIV-1 viral production. (a, b) Higher expression of MRJ-L increases HIV-1 replication. Macrophages derived from six healthy donors (from the first cohort study described in Fig. 1) with low or high expression levels of MRJ-L were infected with HIV-1 at (a) high MOI (p24 = 5 ng/mL) and (b) low MOI (p24 = 50 pg/mL), respectively. Virus output, measured by p24 levels in culture supernatant using ELISA, at day 4 and day 8 after the infection.

The above findings reveal a strong correlation between MRJ-L levels and odds of HIV-1 infection. To investigate the possibility of a causal relationship, we compared the amount of viral replication in macrophages with high MRJ-L levels to that in macrophages with low MRJ-L levels. The cells were infected with equal inocula of either high MOI (Fig. 3a) or low MOI (Fig. 3b) of the macrophage-tropic (M-trophic) HIV-1 ADA strain (Khanna et al., 2000). Interestingly, macrophages with high MRJ-L levels consistently harbored higher levels of HIV-1 replication than those with low MRJ-L levels (Fig. 3a). In the low MOI group, only macrophages with high, but not low, MRJ-L levels showed detectable HIV infection (Fig. 3b).

### 3.4 Depleting MRJ-L Reduces HIV-1 Infection in Macrophages

**Fig. 4:**
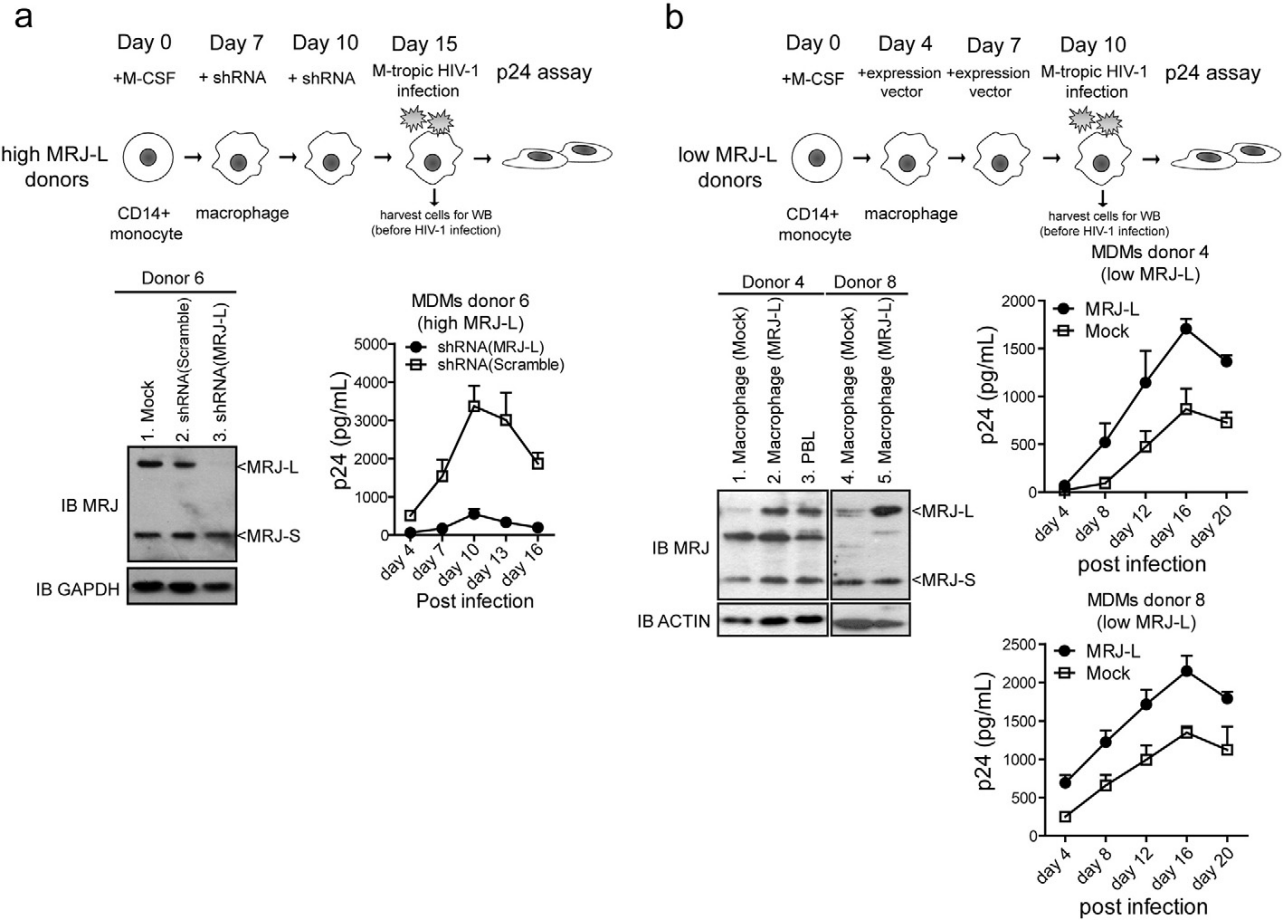
Expression of MRJ-L contributes to HIV-1 viral production. (a) (upper panel) Schematic diagram describing the experimental strategy for lentiviral shRNA depletion of MRJ-L in macrophages derived from monocytes of high MRJ-L healthy donors. The transduction efficiency was about 50–70% as evidenced by a GFP-expressing vector control (Supplementary Fig. 1). (Lower left) Knock down of endogenous MRJ-L in monocyte-derived macrophages (MDMs) from a healthy donor (donor 6) examined by western blotting. Cells were harvested at day 8 after the shRNA treatment. GAPDH was used as a loading control. (Lower right) Virus output in MDMs (donor 6) treated with MRJ-L-shRNA measured by p24 ELISA at days 4, 7, 10, 13, and 16 post-HIV-1 (M-tropic) infection. Data shown represent mean ± SD from triplicate assays. (b) (upper panel) Schematic diagram describing the experimental strategy for lentiviral overexpression of MRJ-L in macrophages derived from monocytes of low MRJ-L healthy donors. (Lower left) Expression profile of MRJ-L by western blot. Cells were harvested at day 6 after vector transduction. (Lower right) Virus output in MDMs (donor 4 and donor 8) overexpressing MRJ-L by p24 ELISA at days 4, 8, 12, 16, and 20 post-HIV-1 (M-tropic) infection.

Based on the above results, we reasoned that siRNA-knock down of MRJ-L should reduce HIV-1 replication. To address this, we knocked down MRJ-L in macrophages with normally high expression levels (from donor 6) (Fig. 4a, western blot, left). Indeed, knock down of MRJ-L significantly reduced HIV-1 infection (Fig. 4a, virus replication curves, right). Next, we examined how exogenous MRJ-L expression would affect virus production in macrophages that naturally have low levels of this protein. The macrophages from donors 4 and 8 contained low levels of endogenous MRJ-L. We transduced these cells with a lentiviral MRJ-L expression vector to raise their MRJ-L levels (Fig. 4b, western blot, left). This increased MRJ-L expression enhanced HIV-1 replication (Fig. 4b). Thus, our results suggest that higher MRJ-L expression pivotally augments HIV-1 infection in macrophages.

### 3.5 MRJ-L Interacts With Vpr and is Required for Its Nuclear Entry

**Fig. 5:**
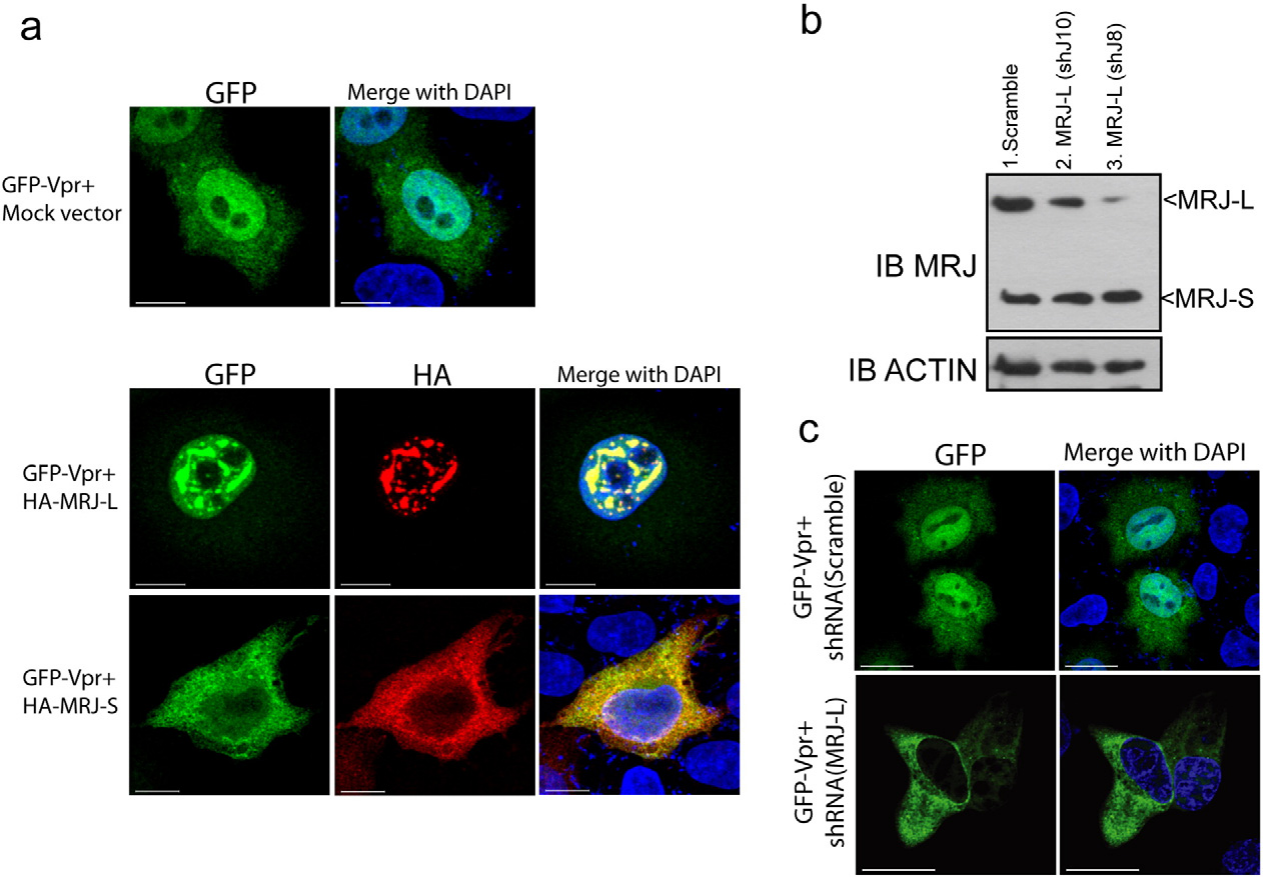
MRJ-L but not MRJ-S mediates nuclear import of Vpr. (a) Localization of Vpr (tagged with GFP) co-expressed with HA-tagged MRJ-L or MRJ-S. Cells were fixed and stained with an anti-HA antibody. Bars: 10 μm. (b) Depletion of MRJ-L in HeLa cells by shRNAs shJ10 or shJ8 was verified by western blotting. > 90% knockdown efficiency was achieved by shJ8. (c) Subcellular localization of Vpr (tagged with GFP) in HeLa cells treated with shRNA (Scramble) or shRNA (MRJ-L, shJ8). Vpr was not detected in the nucleus when MRJ-L was depleted. DAPI staining shows the nuclei. Bars: 10 μm.

Vpr has been implicated in the nuclear import of the HIV-1 pre-integration complex. Because MRJ is a molecular chaperone, we next examined if MRJ-L plays a role in assisting Vpr with nuclear entry. To do this, we co-expressed GFP–Vpr and HA-tagged MRJ-L or MRJ-S in HeLa cells. We observed that Vpr and MRJ-L co-localized in the nuclei of cells while Vpr and MRJ-S were seen in the cytoplasm (Fig. 5a). GFP–Vpr was found exclusively (100%, 30 out of 30) in the nucleus in shRNA (Scramble) cells. When MRJ-L was knocked down in HeLa cells (Fig. 5b, c), the majority (83%, 39 out of 47) of transfected GFP–Vpr became cytoplasmic rather than nuclear [Fig. 5c; compare shRNA (MRJ-L) panels to shRNA (Scramble) panels]. These results indicate that MRJ-L facilitates the nuclear localization of Vpr, a finding consistent with results reported previously on the interaction of MRJ-L with HIV-2 Vpx (Cheng et al., 2008).

## 4 Discussion

Many cellular factors participate in the replication of a virus. A better understanding of the cellular factors involved in viral infection can lead to better antiviral strategies, especially for a difficult virus like HIV-1. We are particularly interested in cellular factors that exhibit different expression patterns between individuals, indicating the presence of natural polymorphisms and which could therefore be amenable to manipulation without grave adverse effects. Vpr is an important auxiliary gene of HIV-1 with multiple functions (Yedavalli et al., 2005, Kogan and Rappaport, 2011). Interestingly, we found that MRJ-L, a cellular protein interacting with Vpr, is enriched when monocytes differentiate into macrophages and has varying expression levels among macrophages from different individuals.

Macrophages play a unique role in HIV-1 infection; among all the quasi-species of HIV-1 in an infected individual, macrophage-tropic strains are preferentially transmitted to a new host (van't Wout et al., 1994, Zhu et al., 1993). When HIV-1 is transmitted to a new host at the mucosal surface, the virus has to undergo ample local expansion to enable dissemination to local lymphoid tissues, or the infection is aborted (Haase, 2010). As macrophages are the primary targets of HIV-1, the transmitted virus should replicate efficiently in macrophages. For non-dividing cells such as macrophages, delivery of the HIV-1 pre-integration complex to the nucleus is a critical and likely rate-limiting step (Segura-Totten and Wilson, 2001). Vpr is primarily responsible for this nuclear transport, and the presence of sufficient MRJ-L protein appears to be essential for Vpr to perform this function.

The distribution of MRJ-L levels seemed to be bimodal in uninfected general population (Figs. 1c and 2). This observation implicated that the expression levels of MRJ-L in macrophages may be regulated by genetic or epigenetic processes, which require future investigation. Furthermore, in vitro infection of HIV-1 for 8 days did not affect MRJ-L level in primary macrophages (Fig. 1d). This evidence strongly suggests that MRJ-L levels should be independent of HIV-1 infection.

Interestingly, macrophages from HIV-1-infected patients had significantly higher expression of MRJ-L compared with macrophages from uninfected individuals. We believe that subjects with lower levels of MRJ-L are less vulnerable to HIV-1 infection because their macrophages cannot support efficient HIV-1 replication. These individuals may require repeated exposure, larger inoculums, or more efficient routes of exposure in order to become infected. In contrast, subjects with high levels of MRJ-L are more susceptible to HIV-1 infection. Hence, people with high levels of macrophage MRJ-L are highly represented in the HIV-1-infected population.

MSM, especially those who practice RUAI, are known to be at particularly high risk for HIV-1 infection (Silan et al., 2013). Our results show that high levels of MRJ-L in macrophages (41.5 fold) are as strong a risk factor as anal intercourse (43.9 fold) for HIV-1 acquisition.

MRJ has two isoforms, a large form (MRJ-L or DnaJB6) and a small form (MRJ-S) (Hanai and Mashima, 2003). The two variants are likely products of alternatively spliced mRNAs. The large form, MRJ-L, is 326 amino acid long and possesses a nuclear localization signal, while the small MRJ-S lacks the carboxyl-terminal 95 amino acids of MRJ-L but contains an additional of 10 amino acids (KEQLLRLDNK). Despite the differences at the carboxyl terminus, both isoforms share similar structures with a conserved J domain (70 amino acids) and a glycine/phenylalanine domain (Liberek et al., 1991). MRJ-S mainly localizes to the cytoplasm while MRJ-L distributes both to the cytosol and the nucleus (Cheetham et al., 1992). It has been suggested that the conserved DNAJ domain was responsible for HSP40's ability to inhibit HIV-1 production (Urano et al., 2013). Our results show that a significant correlation is found between levels of macrophage MRJ-L expression and levels of HIV-1 infection among high-risk subjects. Therefore, natural variation in MRJ-L levels in macrophages and unprotected sex behavior may be the major determinants of susceptibility to HIV-1 infection.

Our collective results suggest that individuals with high levels of MRJ-L may be more susceptible to HIV-1 infection than individuals with low levels of MRJ-L. We demonstrated that MRJ-L interacts with HIV-1 Vpr and assists with HIV-1 replication. A rise in MRJ-L levels effectively increases the replication of HIV-1 and a reduction in MRJ-L expression significantly decreases HIV-1 production. Therefore, the variation in MRJ-L expression in macrophages among different individuals results in differing susceptibilities to HIV-1 infection. In conclusion, strategies to lower MRJ-L levels in macrophages may be beneficial in controlling HIV-1 infection.

## Author Contributions

LMH designed the study. YPC, WHS, PLS, and YHC performed the experiments. HMS executed the yeast two-hybrid experiment. WHS, CCH, SCC, SWH, and YMC recruited HIV-1 patients. YHC, WHS, YPC, PLS, and LMH wrote the manuscript. All authors discussed the results and edited the manuscript.

## Declaration of Interests

All authors declare that they have no competing interests.

## Role of the Funding Source

This project was funded by the National Health Research Institutes, Taiwan (NHRI-EX100-10053SI) and supported by an A1 project grant (97-A109 Establishment of Molecular Virology Lab) from the National Taiwan University Hospital. The funding sources had no involvement in the design, collection, analysis, interpretation, writing, or decision to submit the article.

## Figures and Tables

**Table 1:** Demographic characteristics and MRJ-L expression levels of HIV-1 positive and negative participants.

	HIV-1 (+)	HIV-1 (−)	Total
N = 20	N = 30	N = 50
n (%)	n (%)	n (%)
*Role and protection during anal intercourse*			
Exclusively insertive with condom	0	4 (13)	4 (8)
Exclusively insertive without regular condom	0	3 (10)	3 (6)
Exclusively receptive/versatile with condom	6 (30)	9 (30)	15 (30)
Exclusively receptive/versatile without regular condom	12 (60)	10 (33)	22 (44)
Oral sex	0 (0)	3 (10)	3 (6)
NA	2 (10)	1 (3)	3 (6)

*History of having sexually transmitted diseases*			
No	8 (40)	24 (80)	32 (64)
Yes	2 (10)	0 (0)	2 (4)
NA	10 (50)	6 (20)	16 (32)

*MRJ-L/MRJ-L plus MRJ-S*^a^			
Low (≤ 0.25)	1 (5)	14 (47)	15 (30)
Medium (0.25–0.4)	12 (60)	14 (47)	26 (52)
High (≥ 0.4)	7 (35)	2 (7)	9 (18)

a The MRJ-L expression levels were defined as the ratio of MRJ-L divided by MRJ-L plus MRJ-S.

## Supplementary Data

Supplementary Table 1 Demographic data of HIV-1 positive and negative participants from commercial MSM community.

Supplementary Table 2 Risk factors for HIV-1 infection were analyzed by using Bayesian multiple logistic regression.

Supplementary Fig. 1 Lentiviral transduction efficiency in primary MDMs.
