# Supplementary material for: Large Isoform of Mammalian Relative of DnaJ is a Major Determinant of Human Susceptibility to HIV-1 Infection
Source: eBioMedicine. 2014 Oct 7;1(2-3):126–32. doi: 10.1016/j.ebiom.2014.10.002 (PMC4457413; doi:10.1016/j.ebiom.2014.10.002)
Supplement: Supplementary Table 1 — Demographic data of HIV-1 positive and negative participants from commercial MSM community. [file mmc1.pdf]

Supplemental Information

Supplementary Materials and Methods

Production of lentivirus-packed target genes

293T cells (2×10^6) were transfected using Lipofectamine 2000 (Life Technologies) with 0·45 μg pCMV-ΔR8.91, 0·05 μg pMD.G, and 0·5 μg MRJ-L shRNA vector or a MRJ-L expression plasmid to generate pseudotyped lentiviruses carrying the corresponding genes. Cellular supernatants were harvested at 72 h post-transfection. Viral titer was determined using the protocol provided by the RNAi Core Facility, Academia Sinica, Taiwan (http://rnai.genmed.sinica.edu.tw/protocols).

Fluorescence Confocal Imaging

HeLa cells were co-transfected with pEGFP-Vpr and pcDNA-HA-MRJ expression vectors on glass cover slides. After 24 h, cells were fixed with ice-cold acetone for 10 min and stained with anti-HA antibody (Roche, Palo Alto, CA, USA) for 1 h, washed three times with PBS, and subsequently incubated with AlexaFluor-conjugated anti-mouse antibody for 30 min. After washing two times with PBS, cells were stained with 3 μM 4', 6'-diamidino-2-phenylindole (DAPI) at room temperature for 5 min and

observed under confocal microscopy (Leica TCS SP2).

Supplementary Tables

Supplementary Table 1

Demographic data of HIV-1 positive and negative participants from commercial MSM community.

Variable	HIV-1 (+) N=20 n(%)	HIV-1 (-) N=30 n(%)	Total N=50 n(%)	P value
Age				0.079
<30	12 (60)	19 (63)	31 (62)	
≥30	8 (40)	6 (20)	14 (28)	
NA	0 (0)	5 (17)	5 (10)	
Sexual orientation				0.440
Heterosexual	1 (5)	1 (3)	2 (4)	
Homosexual	18 (90)	29 (97)	47 (94)	
Bisexual	1 (5)	0 (0)	1 (2)	
NA	0 (0)	0 (0)	0 (0)	
Marital status				0.594
Single	17 (85)	27 (90)	44 (88)	
Married	0 (0)	0 (0)	0 (0)	
Divorced/Separated/Widowed	3 (15)	3 (10)	6 (12)	
Education				0.768
<Junior high school	0 (0)	0 (0)	0 (0)	
≥Senior high school	19 (95)	29 (97)	48 (96)	
NA	1 (5)	1 (3)	2 (4)	
Occupation				0.464

Student	4 (20)	8 (27)	12 (24)	
Government employees	5 (25)	3 (10)	8 (16)	
Office worker	7 (35)	13 (43)	20 (40)	
Professional	2 (10)	2 (7)	4 (8)	
Unemployed/ Other	1 (5)	4 (13)	5 (10)	
NA	1 (5)	0 (0)	1 (2)	
Number of sexual partners				0.116
1	5 (25)	5 (17)	10 (20)	
2-3	9 (45)	22 (73)	31 (62)	
≧4	1 (5)	2 (7)	3 (6)	
NA	3 (15)	0 (0)	3 (6)	
Role and protection during anal intercourse				0.07
Exclusively insertive with condom	0	4 (13)	4 (8)	
Exclusively insertive without regular condom	0	3 (10)	3 (6)	
Exclusively receptive / Versatile with condom	6 (30)	9 (30)	15 (30)	
Exclusively receptive / Versatile without regular condom	12 (60)	10 (33)	22 (44)	
Oral sex	0 (0)	3 (10)	3 (6)	

NA	2 (10)	1 (3)	3 (6)	
Times of sexual contact per week				0.364
<1	3 (15)	9 (30)	12 (24)	
1-3	7 (35)	12 (40)	19 (38)	
>3	4 (20)	2 (7)	6 (12)	
NA	6 (30)	7 (23)	13 (26)	
Recreational drug usage				0.451
No	9 (45)	17 (57)	26 (52)	
Yes	8 (40)	7 (23)	15 (30)	
NA	3 (15)	6 (20)	9 (18)	
Oil- or petroleum-based lubricants during sexual intercourse				0.536
Saliva	8 (40)	14 (47)	22 (44)	
Oil-based[a]	6 (30)	5 (17)	11 (22)	
NA	6 (30)	11 (37)	17 (34)	
History of having sexually transmitted diseases				0.012
No	8 (40)	24 (80)	32 (64)	
Yes	2 (10)	0 (0)	2 (4)	
NA	10 (50)	6 (20)	16 (32)	
Frequency of lubricant usage				0.719
Always	14 (70)	24 (80)	38 (76)	
Frequently/Occasionally/ Rarely/Never	3 (15)	3 (10)	6 (12)	
NA	3 (15)	3 (10)	6 (12)	

NA, not available.

Supplementary Table 2

Risk factors for HIV-1 infection were analyzed by using Bayesian multiple logistic regression.

	Odds Ratio	2.5% credible interval	97.5% credible interval
MRJ levels			
MRJ Low	1		
MRJ Medium	7.601	1.37	69.25
MRJ High	41.57	4.285	705.7
Role of sexual behavior			
Insertive anal sex	1		
Receptive anal sex	43.99	2.681	2957
Oral sex	0.2145	8.58E-05	73.13

Supplementary Figure 1

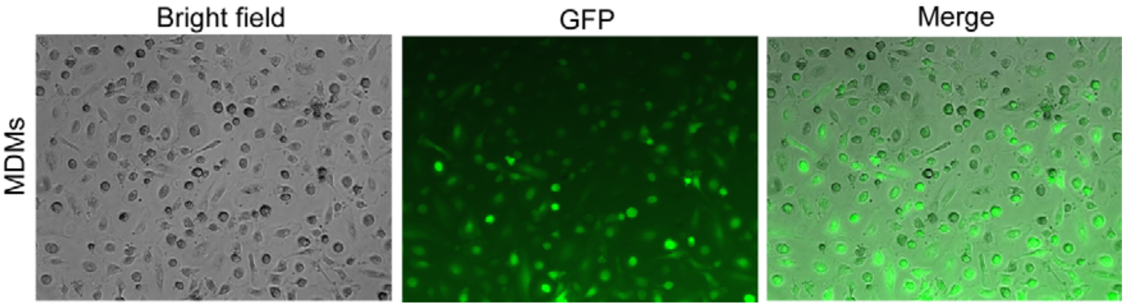

Supplementary Fig. 1. Lentiviral transduction efficiency in primary MDMs. MDMs at day 7 of differentiation were transduced with lentivirus packed with GFP mRNA. The transduction efficiency was about 50-70% as measured by the percentage of cells expressing GFP.
